# Investigating the Influence of High-Speed Gantry Rotation in Cardiac CT on Motion Artifacts in Aortic Stenosis Patients Not Premedicated with β-Blockers: The FAST-CCT Randomized Trial Protocol

**DOI:** 10.3390/jcdd10100424

**Published:** 2023-10-12

**Authors:** Guillaume Fahrni, Giuseppe Gullo, Aisha Touray, Stéphane Fournier, Anne-Marie Jouannic, Henri Lu, Damien Racine, Olivier Muller, Chiara Pozzessere, Salah D. Qanadli, David C. Rotzinger

**Affiliations:** 1 Department of Diagnostic and Interventional Radiology, Lausanne University Hospital and University of Lausanne, Rue du Bugnon 46, 1011 Lausanne, Switzerland; giuseppe.gullo@chuv.ch (G.G.); aisha.touray@chuv.ch (A.T.); anne-marie.jouannic@chuv.ch (A.-M.J.); chiara.pozzessere@chuv.ch (C.P.); 2 Department of Cardiology, Lausanne University Hospital and University of Lausanne, Rue du Bugnon 46, 1011 Lausanne, Switzerland; stephane.fournier@chuv.ch (S.F.); henri.lu@chuv.ch (H.L.); olivier.muller@chuv.ch (O.M.); 3 Institute of Radiation Physics, Lausanne University Hospital and University of Lausanne, Rue du Grand-Pré 1, 46, 1007 Lausanne, Switzerland; damien.racine@chuv.ch; 4 Riviera-Chablais Hospital, Rte du Vieux Séquoia 20, 1847 Rennaz, Switzerland; salah.qanadli@hopitalrivierachablais.ch; 5 Faculty of Biology and Medicine (FBM), University of Lausanne, 1015 Lausanne, Switzerland

**Keywords:** CCTA, motion artifacts, aortic stenosis, gantry rotation speed, 0.23, beta-blockers, high-speed, TAVI, protocol

## Abstract

Background: Coronary CT angiography (CCTA) is increasingly used as a non-invasive tool to assess coronary artery disease (CAD). However, CCTA is subject to motion artifacts, potentially limiting its clinical utility. Despite faster (0.35 and 0.28 s/rot) gantry rotation times, low (60–65 bpm) heartbeat is recommended, and the use of β-blockers is often needed. Technological advancements have resulted in the development of faster rotation speeds (0.23 s/rot). However, their added value in patients not premedicated with β-blockers remains unclear. This prospective single-center, two-arm, randomized, controlled trial aims to assess the influence of fast rotation on coronary motion artifacts, diagnostic accuracy of CCTA for CAD, and patient safety. Methods: We will randomize a total of 142 patients aged ≥ 50 scheduled for an aortic stenosis work-up to receive CCTA with either a fast (0.23) or standard (0.28 s/rot) gantry speed. Primary outcome: rate of CCTAs with coronary motion artifacts hindering interpretation. Secondary outcomes: assessable coronary segments rate, diagnostic accuracy against invasive coronary angiography (ICA), motion artifact magnitude per segment, contrast-to-noise ratio (CNR), and patient ionizing radiation dose. The local ethics committee has approved the protocol. Potential significance: FAST-CCT may improve motion artifact reduction and diagnosis quality, thus eliminating the need for rate control and β-blocker administration. Clinicaltrials.gov identifier: NCT05709652.

## 1. Introduction

Cardiovascular disease, one of the foremost contributors to global mortality and morbidity, results in one person dying every 36 s in the United States alone [1]. Notably, coronary artery disease is the most prevalent type among various cardiovascular conditions [2].

### 1.1. Role and Challenges of CCTA

When assessing coronary artery disease, clinicians have several diagnostic options with different risks and degrees of invasiveness. Coronary catheterization with invasive coronary angiography (ICA) is the current gold standard and has the advantage of combining both diagnosis and therapy (coronary revascularization) within a single session [3]. However, not all patients with suggestive symptoms need a revascularization procedure, as other treatment options exist, such as lifestyle modifications, medical treatment, or surgery. Therefore, assessing coronary artery disease (CAD) with less invasive procedures can be beneficial, sending only selected patients to ICA and revascularization therapy [4]. In this context, different non-invasive tests can help diagnose flow-limiting atherosclerosis, including echocardiography, electrocardiogram, nuclear medicine tests, MRI, and CT. Of those, CT has recently gained interest due to ongoing technological advances [5], with several recent trials demonstrating its clinical value [6,7,8]. One major challenge with coronary CT angiography (CCTA) imaging, unlike extra-cardiac CT imaging, is that coronary arteries are small structures situated within the fast-moving heart, continuously affected by heartbeats. Without a technology to optimize the speed of anatomical sampling and, eventually, motion correction techniques, movement-induced distortion would critically undermine coronary artery analysis [9]. For CCTA to be feasible, images must be acquired using ECG gating synchronized with the heartbeats. CCTA image quality has improved dramatically in the last decade; nevertheless, some limitations persist, one of the most prominent being elevated (>65 bpm) or irregular heart rates. For this reason, oral or intra-venous β-blockers (including but not limited to Metoprolol or Esmolol) are frequently used before the examination to slow down and stabilize the heartbeat. While this procedure is generally safe, it can be time-consuming and still has limitations and contraindications, such as severe asthma, aortic stenosis, allergy, atrioventricular block, or severe chronic obstructive pulmonary disease (COPD) [10]. Additionally, when β-blockers are contraindicated, or a low and regular heart rate is not achieved, a wider acquisition window is typically required, increasing the patients’ exposure to ionizing radiation.

### 1.2. Technical Advancements in Cardiac CTA

In this context, manufacturers have developed multiple technical solutions to help achieve motionless images [11]. On the software side, motion correction algorithms (MCA), typically operating in the image domain, prove valuable in improving image quality and interpretability [12]. On the hardware side, one of the most limiting factors is the speed at which images are captured, known as temporal resolution. This speed is mainly determined by the gantry’s rotational velocity, which includes the tube and detectors of the CT scanner. Images are acquired by rotating the X-ray beam around the patient and using a number of tube-detector pairs operating simultaneously. Until recently, the rotation speed typically used in clinical settings ranged from 0.25 to 0.35 s/rot due to the challenges associated with increasing the rotation speed. Some of the main challenges of accelerating the gantry include the following:

(a) Mechanical stress. The gantry, including the X-ray tube, detectors, and other components, must be designed and built to withstand the increased rotational forces and vibrations associated with higher speeds. This requires robust engineering and precision manufacturing to ensure the gantry can handle the increased load without compromising performance or longevity.
(b) Heat dissipation. Faster rotation times mean shorter exposure times for each angular view, resulting in higher X-ray tube power requirements to maintain image quality. This increased power generation leads to higher heat generation, and effective heat dissipation becomes crucial. Designing tubes that can deliver higher currents and equipped with efficient cooling mechanisms to dissipate the excess heat and prevent overheating is essential for maintaining optimal system performance and preventing damage.
(c) Data acquisition and processing. Faster rotation speeds generate a larger amount of data in a shorter period of time. This increases demand on data acquisition and processing systems, including detector speed, readout capabilities, data transfer rates, and computational power. The system must handle the increased data volume and process it efficiently to reconstruct high-quality images within the desired time frame.

Most recent technical advances overcoming these physical constraints due to acceleration led to faster rotation speeds, up to 0.23 s/rot.

## 2. Materials and Methods

### 2.1. Rationale, Aim, and Design of the Study

This prospective study is conducted in a single Swiss tertiary hospital. It aims to evaluate whether faster gantry rotation speeds in coronary imaging (FAST-CCT) improve the rate of diagnostic images and reduce motion artifacts in patients referred for aortic valve stenosis evaluation. Our institution’s routine protocol for cardiac CT in the work-up of aortic stenosis utilizes a gantry speed of 0.28 s/rot.

Fast gantry rotation CCTA is performed using a 256-multi-detector Revolution^TM^ Apex CT system (GE Healthcare, Waukesha, WI, USA), with gantry rotation speeds of either 0.23 (fast) or 0.28 s/rot (standard). Acquisition and reconstruction parameters are detailed in Table 1. 

**Figure 1:**
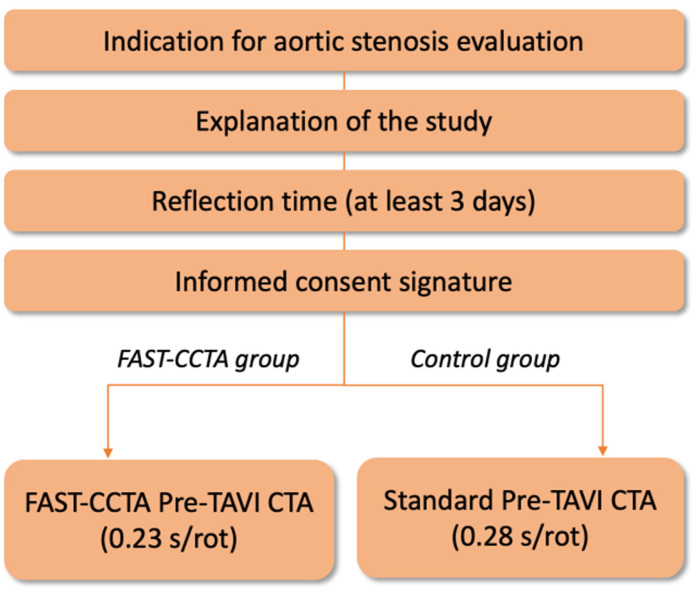
Study flowchart.

For this purpose, a monocentric prospective randomized controlled trial with two arms is designed. The study flowchart is provided in Figure 1. 

### 2.2. Sample Size, Inclusion, and Exclusion Criteria

We conducted a power analysis based on the assumption that standard CCTA in patients undergoing aortic stenosis treatment yields 70% analyzable vessels as previously published [13], and fast rotation CCTA is supposed to provide an improvement in the order 20%, i.e., 90% analyzable segments [14]. The sample size analysis showed that in order to achieve 80% power under the assumed diagnostic CCTA rates, the study should include 62 participants in each arm. Considering an expected weighted kappa of 0.82 [15] for inter-observer variability in the qualitative scoring of vascular motion, the adjusted sample size was 71. Consequently, we intend to have 142 participants with complete datasets.

Inclusion criteria: Participants will be patients holding a clinical indication to undergo cardiac CT due to known or suspected aortic valve stenosis. Participants will be over 50 years old and have signed an informed consent form. 

Exclusion criteria: Patients unable to hold their breath, deaf, or incapable of discernment. Estimated glomerular filtration rate (eGFR) of <30 mL/min/1.73 m^2^. Severe allergy to iodinated contrast medium, manifest thyrotoxicosis. Presence of acute conditions including hemodynamic instability or cardiogenic shock, acute pulmonary edema, exacerbated chronic obstructive pulmonary disease, acute coronary syndrome, pulmonary embolism, or aortic syndrome. Pregnant and breast-feeding women. Patients with prior coronary artery bypass grafting (CABG). Patient psychiatric or psychical conditions undermining the ability to understand study procedures and provide informed consent. Vulnerable subjects.

### 2.3. Characteristics of Participants, Randomization, and Blinding

Most patients screened for aortic stenosis are septuagenarians, even in lower-risk trials [16]. Women of childbearing age are unlikely to undergo aortic stenosis work-up. For this reason, we set the minimum age for eligibility at 50 years. The study population is limited to subjects scheduled to undergo transcatheter aortic valve implantation (TAVI) or surgical aortic valve replacement because (a) CT-based coronary artery analysis is not required in the standard of care during aortic stenosis assessment since patients undergo ICA, and (b) CT is performed without β-blockers. 

Randomization will be performed using the sequentially numbered, opaque sealed envelopes (SNOSE) method with a 1:1 allocation. The randomization list will be electronically generated using R (The R Foundation for Statistical Computing Platform, version 4.1.2) and placed in envelopes before the first patient is included.

The study will be single-blinded to minimize biases for the radiologist reading the images for the study’s purpose. The radiologists reporting the CT for clinical needs (aortic valve assessment) will not be blinded to the assigned group. 

### 2.4. Processes and Interventions

Patients are enrolled as follows. Each time a cardiac CT appointment for aortic stenosis assessment is required, the investigators will contact the patient unless the inclusion/exclusion criteria are unmet. All patients potentially eligible for the study will be approached by phone. The investigators will comprehensively explain the study type, its purpose, CT examination specifics, planned study duration, and anticipated risks and benefits. Interested patients will be sent the patient information sheet via email or regular mail, allowing them ample time to make an informed decision regarding their participation in the investigation. On day 0, subjects will have the opportunity to ask any questions about the study, and those willing to participate will proceed to sign the informed consent sheet. Subsequently, after randomization, the participants will undergo cardiac CT with or without FAST-CCT based on their assigned group.

### 2.5. Measured Outcomes

**Figure 2:**
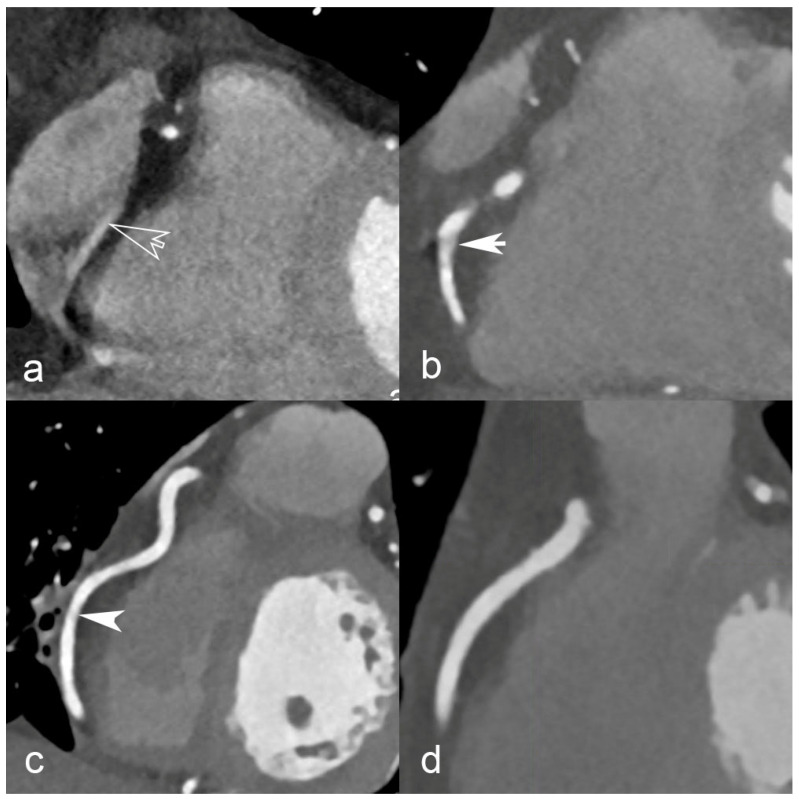
Examples of coronary CT angiography images illustrating the range of motion artifact magnitudes. Each panel corresponds to a qualitative rating on a 4-point scale: (**a**) poor, open-headed arrow indicates severe artifacts impairing accurate evaluation, segment classified as non-evaluable; score = 1, (**b**) adequate, white arrow indicates moderate artifacts, acceptable for routine clinical diagnosis; score = 2, (**c**) good, white arrowhead indicates minor artifacts, good diagnostic quality; score = 3, and (**d**) excellent, no artifacts; score = 4.

The primary endpoint of this study will be to evaluate the rate of CCTAs with coronary motion artifacts hindering interpretation in patients undergoing cardiac CT before aortic stenosis treatment, with fast (0.23 s/rot) vs. standard (0.28 s/rot) rotation speed. Two radiologists blinded towards the gantry rotation speed will assess motion artifacts on coronary segments with a diameter of at least 1.5 mm, using an ordinal scale as previously published [17]: “excellent (no artifacts; score = 4), good (minor artifacts, good diagnostic quality; score = 3), adequate (moderate artifacts, acceptable for routine clinical diagnosis; score = 2), or poor (severe artifacts impairing accurate evaluation, segment classified as non-evaluable; score = 1)”. Examples of each qualitative motion artifact rating score are provided in Figure 2. The rate of CCTAs with coronary motion artifacts hindering interpretation will thus be defined as (CCTAs with any segment score = 1/total CCTAs). The rationale behind the choice of this primary endpoint is that only CCTAs without motion artifacts can provide clinical value.

The secondary endpoints (comparison between arms) will be the following: difference assessable coronary segments rate; diagnostic accuracy against ICA; motion artifact magnitude per segment; quantitative image quality measured with contrast-to-noise ratio (CNR); patient ionizing radiation dose; TAVI sizing efficacy. Assessable coronary segments will be defined with the same score as the primary endpoint. For the diagnostic accuracy against ICA endpoint, coronary stenoses will be assessed and compared with stenoses values from the ICA reports. TAVI sizing efficacy will be assessed by evaluating motion artifacts as either present or absent. These artifacts are defined as an annular double contour with unclearly defined measurement targets for diameter, perimeter, and area. Finally, in the overall study cohort, multivariate analysis will be conducted to assess predictors of unsuccessful CCTA. The rationale behind choosing these secondary endpoints is that they all measure quality (or safety). CNR will be calculated using the following formula:

(1) $$CNR=\frac{\left|{mean\;CT\;number}_{lumen}-{mean\;CT\;number}_{fat}\right|}{\sqrt{\frac{1}{2}({SD}_{lumen}^{2}+{SD}_{fat}^{2})}}$$

where “lumen” is obtained from a region of interest in the coronary lumen, and “fat” is obtained from a region of interest in the pericardial fat.

### 2.6. Data Management 

Trial data is securely stored on a REDCap database, a broadly used HRA-compliant data collection application [18], assuring traceability of data editing with hourly automatic backups (audit trail). Data will be coded and archived for at least 10 years after study termination or premature termination of the study.

### 2.7. Safety Considerations

Patients screened for inclusion will all have a clinical indication for a cardiac and aortic CTA as part of the aortic stenosis work-up. This applies to enrolled participants and those who decline to participate, ensuring an equal administration of intravenous iodinated contrast media and radiation dose for all cases. However, patients enrolled in the fast rotation group may potentially experience lower radiation dose exposure.

Device deficiencies (DDs) and adverse events (AEs), including serious adverse events (SAEs), will be diligently collected, thoroughly investigated, and documented in both the source document and electronic case report form (CRF) maintained on the REDCap throughout the entire investigation period. Patients who agree to participate will not be subjected to any additional procedures or follow-up visits.

### 2.8. Type of Data

The following clinical and laboratory data will be collected: serum creatinine (and estimated glomerular filtration rate); height, weight, body mass index (BMI); dyslipidemia; current or past smoking; hypertension; diabetes; family history of CAD; hypersensitivity reaction to iodinated contrast medium; heart rate before and during CCT acquisition. 

Cardiac CTA and ICA imaging data will be stored electronically in a picture archiving and communication system (PACS).

Radiation exposure data, including dose measurements, will also be collected for patients in both groups.

### 2.9. Statistical Analysis

The null hypothesis postulates no statistically significant difference in the coronary artery non-interpretability rate due to motion artifacts in both study groups. Conversely, the alternative hypothesis suggests a statistically significant difference in the study groups’ coronary artery motion artifact rate.

Statistical analysis will be conducted using R (The R Foundation for Statistical Computing Platform, version 4.1.2). The results will be presented as the number of subjects (percentage), mean (±SD), or median (IQR) for non-normally distributed data unless otherwise specified. Data normality will be assessed using the Shapiro–Wilk test. The rate of diagnostic CCTA in each arm will be compared using the chi-squared test. Additionally, further bivariate statistical analyses will be conducted employing chi-squared, Wilcoxon two-sample, or Student’s *t*-tests where appropriate.

In the overall cohort, factors associated with unsuccessful coronary artery analysis will be assessed through binomial logistic regression, with the following independent variables: gantry rotation speed, heart rate, body mass index (BMI), age, sex, and coronary artery calcium score. The significance level will be two-sided, α = 0.05. Interobserver agreement for qualitative ratings will be evaluated using weighted kappa coefficients and interpreted as follows: ≤0.4, poor; 0.4–0.75, fair to good; >0.75, excellent.

### 2.10. Ethical Considerations and Declarations

The study protocol (version 2, 2 February 2023) was approved by the ethical committee of the Canton de Vaud (CER-VD) on 31 May 2023 (protocol number 2023-D0002). The study complies with the principles of the Declaration of Helsinki and was registered on ClinicalTrials.gov (NCT05709652). Only participants who give written informed consent will be included.

### 2.11. Study Status and Timeline

The study is not open yet. The recruitment is expected to begin in 2023 and be completed by the end of 2025.

## 3. Discussion

### 3.1. Rationale of the FAST-CCTA Study

Technological advances in gantry maximum rotation speed are on the rise. Improved rotation speeds have been shown to result in improved image quality and motion artifacts [19,20,21,22,23,24,25,26]. With a rotation speed of 0.35 s/rot, Kojima et al. defined an upper heart rate limit of 60 bpm for image quality in CCTA with ultra-high-resolution CT [27]. Few studies are focused on the benefit of improved gantry rotation time. In a retrospective study including 160 patients, Beeres et al. found that 0.28 s/rot and 0.33 s/rot reduced motion artifacts but resulted in more streak artifacts, image noise, and reduced image quality for whole chest CT scans [28]. In a preliminary study including 50 consecutive patients with elevated (>70 bpm) heart rates without β-blockers, Belsack and al. found subjective improvements in image quality with 0.23 s/rot [29]. To our knowledge, there are no peer-reviewed published data of prospective studies on the impact of FAST-CCT on coronary motion artifacts in the absence of β-blocker premedication.

This study is anticipated to have a significant clinical impact given the potential improvement in the rate of diagnostic-quality images and the feasibility of performing CCTA without the need for β-blocker premedication. The results are expected to be scientifically robust due to the study’s prospective design, randomization, and power analysis.

### 3.2. Limitations of the Study Design

There might be a bias due to patient selection. As our study includes patients referred for aortic valve stenosis assessment, the population will predominantly consist of elderly patients with a likely high prevalence of coronary artery disease. These patients are more prone to poor image quality than younger patients’ normal coronary arteries. However, this is not a concern, as it would lead to an underestimation, rather than overestimation, of the benefits of improved gantry rotation on image quality. Another limitation of the selected population is the range of heart rates, which may differ from the general population undergoing routine CCTA.

### 3.3. Dissemination

Once terminated, study results will be submitted for publication in a peer-reviewed scientific journal. We intend to communicate the results to the public via scientific media outlets if the results are compelling enough. We will implement substantial amendments only following a formal agreement by the external ethics committee.

## Figures and Tables

**Table 1:** Acquisition and reconstruction parameters. DLIR H = deep learning image reconstruction at high strength.

	Standard Protocol	Fast Protocol
Tube voltage (kVp)	100	100
Tube current (mA)	500–1000	500–1000
Noise index	20	20
Rotation time (s/rot)	0.28	0.23
Acquisition mode	Axial	Axial
Injection phase	Arterial	Arterial
Phases of cardiac cycle	20–80% (prospective)	20–80% (prospective)
Collimation (mm)	256 × 0.625	256 × 0.625
FOV (mm)	220–250	220–250
Matrix size (pixels)	512 × 512	512 × 512
Slice thickness (mm)	0.625	0.625
Slice incrementation (mm)	0.6	0.6
Reconstruction kernel	Standard	Standard
Reconstruction algorithm	DLIR H	DLIR H

## Data Availability

This protocol presents no patient data.
